# Evidence that central pathways that mediate defecation utilize ghrelin receptors but do not require endogenous ghrelin

**DOI:** 10.14814/phy2.13385

**Published:** 2017-08-11

**Authors:** Ruslan V Pustovit, Brid Callaghan, Mitchell T Ringuet, Nicole F Kerr, Billie Hunne, Ian M Smyth, Claudio Pietra, John B. Furness

**Affiliations:** ^1^ Department of Anatomy & Neuroscience University of Melbourne Parkville Victoria Australia; ^2^ Florey Institute of Neuroscience and Mental Health Parkville Victoria Australia; ^3^ Department of Anatomy and Developmental Biology Monash University Clayton Victoria Australia; ^4^ Helsinn Research and Preclinical Department Lugano Switzerland

**Keywords:** Defecation reflex, ghrelin, intestinal innervation

## Abstract

In laboratory animals and in human, centrally penetrant ghrelin receptor agonists, given systemically or orally, cause defecation. Animal studies show that the effect is due to activation of ghrelin receptors in the spinal lumbosacral defecation centers. However, it is not known whether there is a physiological role of ghrelin or the ghrelin receptor in the control of defecation. Using immunohistochemistry and immunoassay, we detected and measured ghrelin in the stomach, but were unable to detect ghrelin by either method in the lumbosacral spinal cord, or other regions of the CNS. In rats in which the thoracic spinal cord was transected 5 weeks before, the effects of a ghrelin agonist on colorectal propulsion were significantly enhanced, but defecation caused by water avoidance stress (WAS) was reduced. In knockout rats that expressed no ghrelin and in wild‐type rats, WAS‐induced defecation was reduced by a ghrelin receptor antagonist, to similar extents. We conclude that the ghrelin receptors of the lumbosacral defecation centers have a physiological role in the control of defecation, but that their role is not dependent on ghrelin. This implies that a transmitter other than ghrelin engages the ghrelin receptor or a ghrelin receptor complex.

## Introduction

Activation of the conventional ghrelin receptor (GHSR1a) causes coordinated propulsive activity of the colorectum and increased defecation in rat and mouse (Shimizu et al. 2006; Charoenthongtrakul et al. 2009). Moreover, GHSR1a agonists increase defecation in human (Ejskjaer et al. 2010; Ellis et al. 2015; Acosta et al. 2016). In rat, experiments using centrally penetrant GHSR1a agonists with different chemistries have shown the site of action to be the defecation center in the lumbosacral spinal cord (Shimizu et al. 2006; Hirayama et al. 2010; Pustovit et al. 2014; Naitou et al. 2015). The effects of intravenous GHSR1a agonists were mimicked by applying ghrelin intrathecally, but responses could not be evoked by intravenous ghrelin, which does not enter the spinal cord (Hirayama et al. 2010). Defecation in response to centrally penetrant agonists was prevented by cutting the pelvic nerves between the defecation center and the colorectum, but not by cutting the spinal cord rostral to the defecation center (Shimizu et al. 2006; Pustovit et al. 2014; Naitou et al. 2015). Consistent with ghrelin agonists, and intrathecal ghrelin, acting at the lumbosacral defecation center, GHSR1a expression was observed in autonomic preganglionic neurons in this spinal cord region by in situ hybridization histochemistry (Ferens et al. 2010a) and in ghrelin receptor reporter mice (Furness et al. 2011).

While these observations imply that GHSR1a is in the pathways of defecation control, ghrelin itself is not found in the mouse spinal cord (Furness et al. 2011). In fact, it is probable that ghrelin is not present even in other parts of the central nervous system (Sakata et al. 2009; Furness et al. 2011; Kern et al. 2014; Cabral et al. 2017).

Despite the apparent absence of ghrelin, the conservation of effect of ghrelin receptor agonists in mouse, rat and human, suggests that GHSR1a receptors in the defecation pathways may have a physiological role. The current experiments were devised to test the proposition that there is a natural stimulant of ghrelin receptors in the defecation center that is released in the spinal cord, but is not ghrelin.

## Materials and Methods

Experiments were conducted on 8–12 weeks old Sprague–Dawley rats and Sprague–Dawley rats in which ghrelin were knocked out using CRISPR technology. Procedures were approved by the University of Melbourne Animal Ethics Committee. Rats were supplied with food and water ad libitum prior to the experiments.

### Ghrelin knockout rats

Ghrelin was knocked out using Cas9/CRISPR gene editing technology by the Australian Phenomics Network CRISPR service, Monash University. Two microinjection sessions were employed using two different guides. In the first microinjection session, guide 5′‐1 GGT was the protospacer adjacent motif (PAM) followed by TGGACTCGTTTAAGATTCCG, and guide 3′‐1 was GCATGCCTGAAAGGGTCTAA with AGG as the PAM. In the second session guide 5′‐2 was GGG(PAM) TGGGAACATTCTACCACTGT and guide 3′‐2 was CTGAGAAATAAGAGCTACAC with TGG as the PAM. The PCR genotyping assay used primers external to the ~4.3 kb region that was deleted and amplified a product of ~550 bp (for guides 5′‐1 & 3′‐1) or ~400 bp (for guides 5′‐2 & 3′‐4) when the correct modification had taken place. Sequencing revealed that two transgenic ghrelin knockout male rats were generated from the first guide pair and two transgenic knockout females and four transgenic knockout males were generated from the second guide pair and these founder animals were transferred to the University of Melbourne for breeding. For genotyping, the progeny of the founder animals, two rounds of PCR were employed; the forward 5′ ATGCATGAACATGTGTGCTCG 3′ and reverse (5′ TAGCCTTGAGCACATGGGAC 3′) genotyping primers amplified a product of approximately 550 bp or 400 bp if the modified allele was present. Rats identified as having a modified allele underwent a second PCR to determine homozygosity or heterozygosity. The primers used were 5′CTGCAGTTTGCTACTCCTCA 3′ and 5′ TGGTGGCTTCTTGGATTCCT 3′generating a band of 689 bp if the rats were heterozygous. For all rats that were used in physiological experiments, the absence of ghrelin was confirmed by immunohistochemistry of gastric sections.

### In vivo experiments

Rats were sedated with ketamine hydrochloride (50–60 mg·kg^−1^, i.m.), following which anesthesia was induced with *α*‐chloralose (60 mg·kg^−1^, i.v.). The femoral artery was then cannulated for the infusion of anesthetic and blood pressure recording, and the femoral vein was cannulated for delivery of drugs. Blood pressure and heart rate were recorded with a Power Lab recording system using Chart 5 software (both from ADInstruments, Sydney, Australia). Anesthesia was maintained by intraarterial infusion of *α*‐chloralose (12–20 mg·kg^−1^·h^−1^) plus ketamine (3–5 mg·kg^−1^·h^−1^) in phosphate‐buffered saline (PBS; 0.15 mol/L NaCl containing 0.01 mol/L sodium phosphate buffer, pH 7.2). The distal colon was cannulated at the colonic flexure, which in the rat is at the junction of the proximal and distal colon, where formed fecal pellets are first observed. A second cannula was inserted into the anus. The colon remained in situ, and the muscle and skin were closed around the proximal cannula. The oral cannula was connected to a Mariotte bottle filled with warm PBS, and the distal cannula to a pressure transducer via a one‐way valve. The baseline intraluminal pressure was maintained at 6–10 mmHg by adjusting the heights of the Mariotte bottle and outlet. Expelled fluid was collected in a cylinder distal to the one‐way valve, and measured by weighing with a force transducer. Blood pressure measurements were made continuously. At the end of each experiment, the rat was killed with a lethal dose of sodium pentobarbitone (300 mg·kg^−1^ i.v.), while still under anesthesia.

### Spinal cord lesion

A total of nine wild‐type rats had their spinal cord severed at T4. The rats were lesioned at 8–10 weeks of age and left for 5 weeks to recover after surgery. A total of 10 sham operations were conducted at the same ages. Spinal transections were made using aseptic conditions under inhaled isoflurane anesthesia (3% in oxygen). The muscles connecting to the spines of the vertebrae were detached and a laminectomy was performed to remove the dorsal aspect of the T4 vertebrum. The spinal cord was raised using a curved probe so that complete transection could be observed. The cord was transected with sharp scissors. The laminectomy was closed with three layers of sutures through the adjacent vertebral musculature, the subdermal tissues and the skin. The area was then disinfected with chlorhexidine in 70% alcohol. Sterile saline (1.5 mL/100 g body weight) and analgesic, buprenorphine (0.03 mg·kg^−1^), were administered postoperatively by subcutaneous injection. After recovery from anesthesia, the animals were examined to confirm paralysis of the hind limbs as an index of the completeness of the lesion. Additional injections of sterile saline and analgesic were administered twice daily for the first 3 days postoperatively. For the first 10–14 days after the spinal cord was transected, until the bladders of spinal cord injury (SCI) animals started voiding spontaneously, prophylactic antibiotic (cefazolin 50 mg·kg^−1^) was injected at 12 h intervals, and bladders of SCI rats were manually expressed by external palpation 2–3 times per day.

### Water avoidance test

Fecal output in response to water avoidance was measured as previously described (Million et al. 2000; Pustovit et al. 2015). Ghrelin knockout rats and their wild‐type littermates were familiarized with handling over a period of 2 weeks. On the experimental day, the rats were injected with the GHSR1a receptor antagonist, YIL781 (Tocris Bioscience, Bristol, UK)*,* 3 mg·kg^−1^, i.p., or vehicle. Ten min later the rat was placed on a platform (height 8 cm, length 6 cm, and width 6 cm), located in the middle of a large plastic tub that was filled with water up to 7 cm (1 cm below the top of the platform), for a period of 60 min. The numbers of fecal pellets produced by the rat in the first 10, 15, 30, 45, and 60 min on the platform were counted. Following completion of WAS experiments, the rats were culled and stomachs collected for confirmation of genotype by immunohistochemistry and numbers of residual pellets were counted.

### Immunohistochemistry

Three Sprague–Dawley wild‐type rats were anesthetized with a mixture of ketamine hydrochloride (100 mg·kg^−1^) and xylazine (20 mg·kg^−1^), both from Troy Laboratories (Sydney, Australia), and a perfusion needle was inserted transcardially into the aorta. The right atrium was cut open and the animal was perfused with heparinized PBS followed with 2% formaldehyde plus 0.2% picric acid in 0.1 mol/L sodium phosphate buffer, pH 7.2. After perfusion was completed, the stomach and lumbosacral spinal cord were removed and post‐fixed in the same fixative overnight at 4°C. Fixative was washed out with 3 × 10 min washes in DMSO followed by 3 × 10 min washes in PBS and then placed in PBS sucrose azide (PBS containing 30% sucrose as a cryoprotectant and 0.1% sodium azide) and stored at 4°C. Tissue was transferred to a mixture of PBS sucrose and optimum cutting temperature (OCT) compound (Tissue Tek, Elkhart, IN, USA) in a ratio of 1:1 for 24 h before being embedded in 100% OCT and sectioned (12 *μ*m thick) on a cryostat.

### Antibodies

Antibodies that were used are listed in Table 1. Antibody RY1601 was raised against synthetic rat acylated ghrelin (1–15)‐Cys, coupled to keyhole limpet hemocyanin (KLH) and antibody RY1595 was raised against synthetic rat desacylated ghrelin (1–15)‐Cys, similarly coupled to KLH (Mizutani et al. 2009). Both antibodies were raised in rabbits. Previously published results show that immunoreactivity using RY1601 was abolished when the antibody was absorbed against ghrelin, but was not absorbed by desacyl ghrelin; conversely RY1595 was absorbed by desacyl ghrelin, but was not absorbed by ghrelin itself (Mizutani et al. 2009). Lack of cross‐reactivity was confirmed by peptide‐specific enzyme linked immunosorbent assay (ELISA) (Mizutani et al. 2009). Antibody GO‐1 was raised in a rabbit against synthetic human acylated ghrelin coupled through glutaraldehyde to bovine serum albumin (Patterson et al. 2005). This antibody has been shown to cross react 100% with human and rat ghrelin, but not with desacyl ghrelin of either species, or any other known gastropancreatic peptide or hormone (Patterson et al. 2005). Radioimmunoassay of fractions separated by fast protein liquid chromatography of plasma samples confirmed that antibody GO‐1 specifically detects acylated ghrelin (Patterson et al. 2005). Chicken anti‐ghrelin (Abcam; ab15861) is a polyclonal antibody raised in chicken against amino acids 24–37 (GSSFLSPEHQKAQQ) of mouse ghrelin. Anti‐neuropeptide Y (NPY; E2210) was raised in a sheep against rat NPY1‐36 coupled to bovine serum albumin. Immunoreactivity revealed by this antibody was eliminated by preincubation with NPY (Furness et al. 1985). Anti‐neuronal NOS was raised in sheep against rat nNOS. Specificity of the antibody is demonstrated by western blot analysis of rat brain tissue, revealing a solid band at ~155 kD, consistent with molecular mass of purified nNOS (Herbison, 1996). Furthermore, immunoreactivity of the antibody was eliminated in western blots following preadsorption with recombinant nNOS protein (Herbison, 1996).

**Table 1:** Primary antisera, their respective hosts, dilutions, reference codes and sources, used in this study

Tissue antigen or label	Host species	Antibody code	Dilution	Source or reference
Acylated ghrelin	Rabbit	RY1601	1:10,000 (stomach); 1:1000 (spinal cord)	Mizutani et al. (2009); Furness et al. (2011)
Acylated ghrelin	Rabbit	G01	1:3000 (stomach); 1:500 (spinal cord)	Patterson et al. (2005); Furness et al. (2011)
Acylated ghrelin	Chicken	Ab15861	1:800 (stomach and spinal cord)	Abcam, Cambridge, UK
Desacyl ghrelin	Rabbit	RY1595	1:10,000 (stomach); 1:1000 (spinal cord)	Mizutani et al. (2009); Furness et al. (2011)
Neuronal NOS (nNOS)	Sheep	V205	1:5000 (stomach and spinal cord)	Williamson et al. (1996)
Neuropeptide Y	Sheep	E2210	1:400 (stomach and spinal cord)	Furness et al. (1985)

**Table 2:** Secondary antisera their respective fluorophores, dilutions and sources, used in this study

Antibody	Fluorophore	Dilution	Source and product code
Donkey anti‐rabbit	Alexa Flour 488	1:1000	Invitrogen, Carlsbad, CA, USA A21206
Donkey anti‐sheep	Alexa Flour 594	1:500	Invitrogen, Carlsbad, CA, USA A11016
Donkey anti‐chicken	Alexa Flour 488	1:500	Jackson Immuno Research Laboratories Inc., West Grove, PA, USA, 703‐545‐155

### ELISA

Rats were anesthetized by placing them in a chamber containing isoflurane. Once the animal was sedated the carotid artery was severed. Stomach, hypothalamus, lower brain stem, and the lumbosacral region of the spinal cord were then dissected from the animal, placed in microfuge tubes, snap frozen in liquid nitrogen and stored at −80°C. Five volumes of sterile dH_2_O (0.5 mL per 100 mg tissue) containing protease inhibitor cocktail (P8340, Sigma‐Aldrich, Sydney, Australia) was added to the tissue and tubes were immediately placed in a floating rack in a beaker of boiling water on a hotplate and boiled for 5 min, then sonicated on ice and centrifuged at 11,000 *g* for 10 min at 4°C to remove cellular debris. The supernatant was collected and further diluted in dH_2_O (wild‐type stomach 1:100, other tissues 1:10) and HCl was added at a final concentration of 0.05 mol/L.

The ghrelin rat/mouse ELISA kit (EZRAGRA‐90K; Millipore, Darmstadt, Germany) was run as per manufacturer's instructions, with 20 *μ*L of diluted tissue sample loaded into wells in duplicate. Prior to acidification, protein concentrations of tissue samples were determined using a Quick Start Bradford Protein Assay (Bio‐Rad Laboratories, Gladesville, Australia), and ghrelin assay values were normalized against total protein concentration to make results comparable.

### Data and statistics

Data are expressed as mean ± SEM. Statistical comparisons are made using Student's *t*‐test or one‐way ANOVA with Tukey's multiple comparisons test, as indicated in the text. Results were considered to differ significantly when *P* ≤ 0.05.

## Results

### Immunohistochemical localization of ghrelin

**Figure 1:**
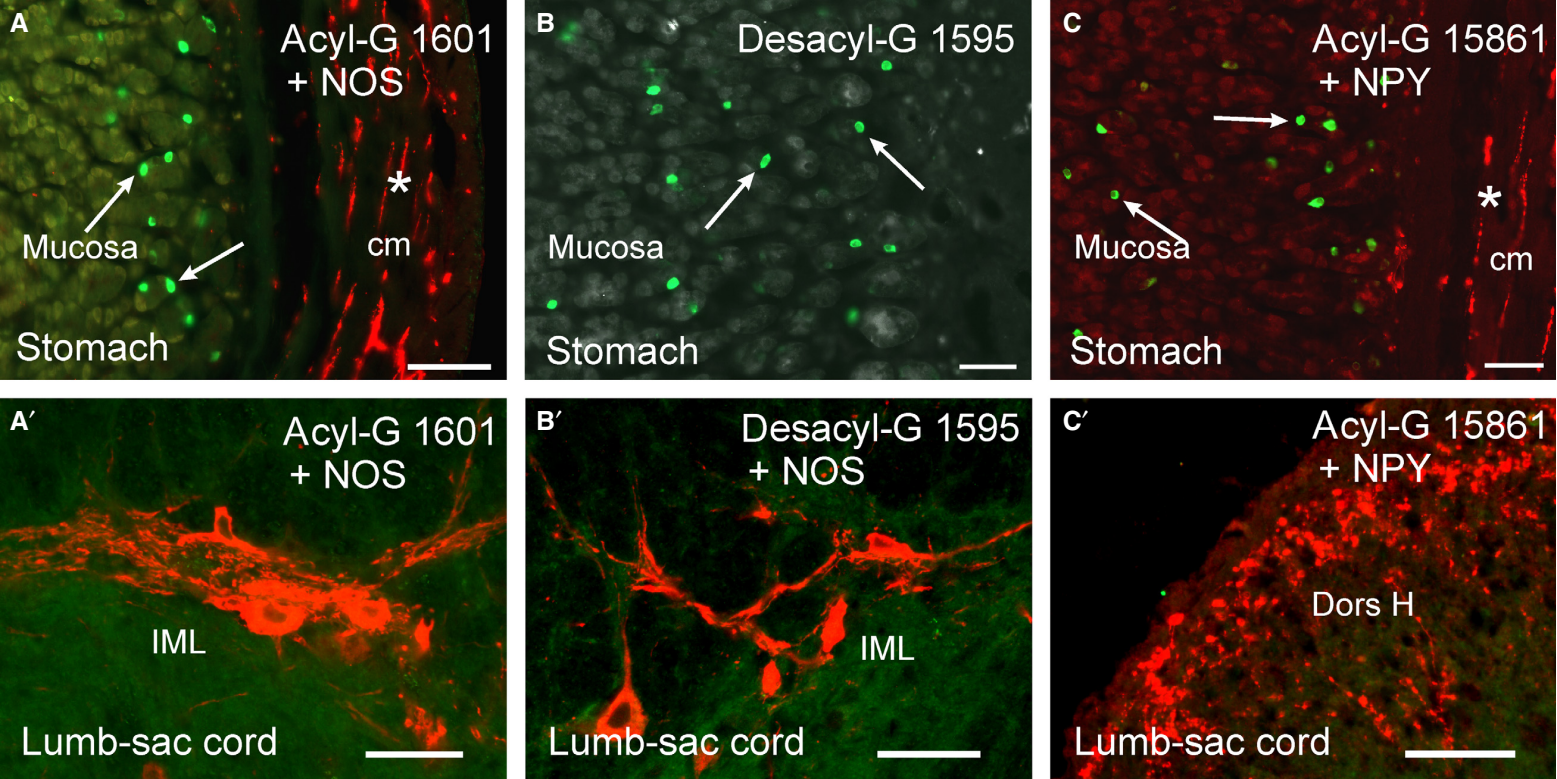
Ghrelin immunoreactivity was revealed in the stomach, but not in the spinal cord, using the same antisera. Anti‐ghrelin antibody, 1601, revealed ghrelin‐containing enteroendocrine cells in the gastric mucosa, indicated by arrows (A). Anti‐neuronal NOS (nNOS) revealed nerve fibers in the circular muscle (cm, nNOS fiber indicated by asterisk) in the same sections. The same combination of antibodies revealed nNOS nerve cells and fibers of the intermediolateral cell columns (IML) in the lumbosacral spinal cord, where ghrelin acts, but no ghrelin immunoreactivity could be found (A′), even when the concentration of antibody was increased. Similar results were obtained with anti‐desacyl ghrelin in the stomach and lumbosacral spinal cord (B, B′), with desacyl ghrelin being revealed in the stomach but not the lumbosacral spinal cord. In C, C′, simultaneous staining for ghrelin and NPY, using anti‐ghrelin antibody 15,861, reveals ghrelin cells in the rat stomach and NPY in nerve fibers of the stomach and the lumbosacral spinal cord. No ghrelin immunoreactivity was detected in the spinal cord. As illustrated in C′, peptide transmitters are concentrated in nerve terminals and are readily visualized. The exposure time for image capture has been set so that the tissue background can be seen in a and b. Scale bars = 50 *μ*m.

Each of the three anti‐acyl ghrelin antibodies and the anti‐desacyl ghrelin antibody revealed ghrelin immunoreactive enteroendocrine cells (EEC) in the rat stomach (Fig. 1). By contrast, in sections of the rat spinal cord, no immunoreactivity for ghrelin was detected. To further investigate this observation, concentrations of antibodies were increased over the concentrations that gave strong immunoreactivity in gastric EEC (RY1601, 10‐fold; G01, 6‐fold; RY1595 10‐fold). The higher concentrations resulted in a diffuse background stain in the white and gray matter of the spinal cord, light, even staining of large ventral horn cells, but no staining of nerve terminals. By contrast, antibodies against a neuropeptide known to be in the spinal cord, NPY, revealed bright immunoreactivity in the dorsal horns and in the intermediolateral cell columns. Neuronal NOS immunoreactivity was used to specifically locate the intermediolateral cell columns where ghrelin ligands act. No acyl ghrelin or desacyl ghrelin terminals were observed in these cell groups (Fig. 1). Double labeling for ghrelin and neuronal NOS and for ghrelin and NPY was also conducted in the stomach. Both were revealed, indicating that localizing neuronal NOS or NPY did not interfere with ghrelin immunoreactivity.

### Ghrelin immunoassay

**Figure 2:**
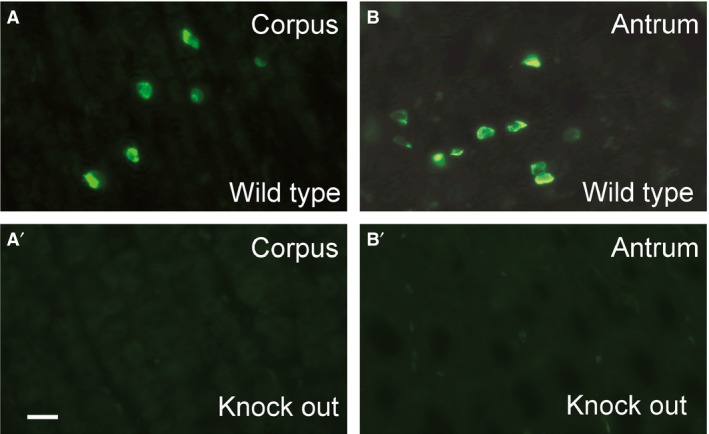
Immunohistochemistry for ghrelin, using anti‐ghrelin antibody 1601, in the mucosa of the gastric corpus and antrum of the wild‐type and ghrelin‐deficient rats. Gastric enteroendocrine cells with ghrelin immunoreactivity were common in the stomachs of wild‐type rats (A, B) but were absent from stomachs of the ghrelin knockout rats (A′, B′). Scale bar = 20 *μ*m (applies to all panels).

Ghrelin immunoreactivity was quantified in extracts of stomach, hypothalamus, lower brain stem (medulla) and lumbosacral spinal cord, from both wild‐type and ghrelin knockout rats, using ELISA. The knockout of ghrelin was investigated by genotyping and by immunohistochemistry for ghrelin in the stomach and CNS. Genotyping showed gene inactivation and immunohistochemistry showed that there was no detectable ghrelin in either the antrum or the corpus in the ghrelin knockout rats (Fig. 2).

**Figure 3:**
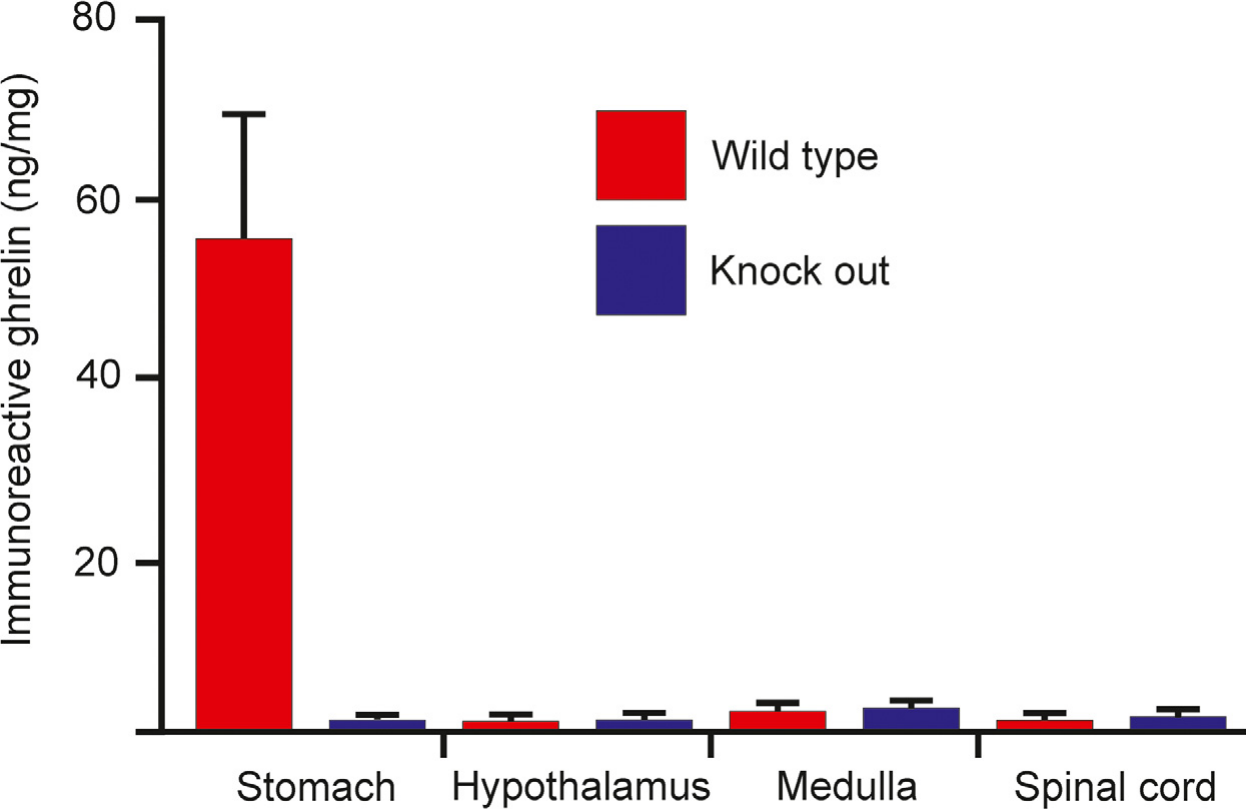
Measurement of ghrelin immunoreactivity in extracts from the rat stomach, brain and spinal cord. The measured ghrelin immunoreactivity in the wild‐type stomach is statistically significantly different from measurements in all other tissue extracts (*P* < 0.05; one‐way ANOVA with Tukey's multiple comparisons test). Differences between measurements in other regions (wild‐type and knockout) are not statistically different. Measurement is of rat ghrelin 1–28 immunoreactive equivalents. The lack of difference between knockout and wild‐type in the CNS indicates that there is a low background binding in this immunoassay.

Immunoassay of extracts from the stomach revealed high concentrations of ghrelin, despite the ghrelin‐containing enteroendocrine cells being a low proportion of the cell types in the stomach. The signal detected by this ELISA was reduced to background levels in gastric extracts from the ghrelin −/− rats, compared to the wild‐type rats (Fig. 3). There were very low levels of binding in the extracts from the hypothalamus, lower brain stem and lumbosacral spinal cord. The small signals in these CNS regions were not different in tissue from ghrelin knockout and wild‐type rats (Fig. 3). This indicates that there is background binding in this immunoassay, as there can also be background binding in immunohistochemistry for ghrelin (Furness et al. 2011).

### Effects of spinal cord transection

#### Fecal release in response to water avoidance stress

**Figure 4:**
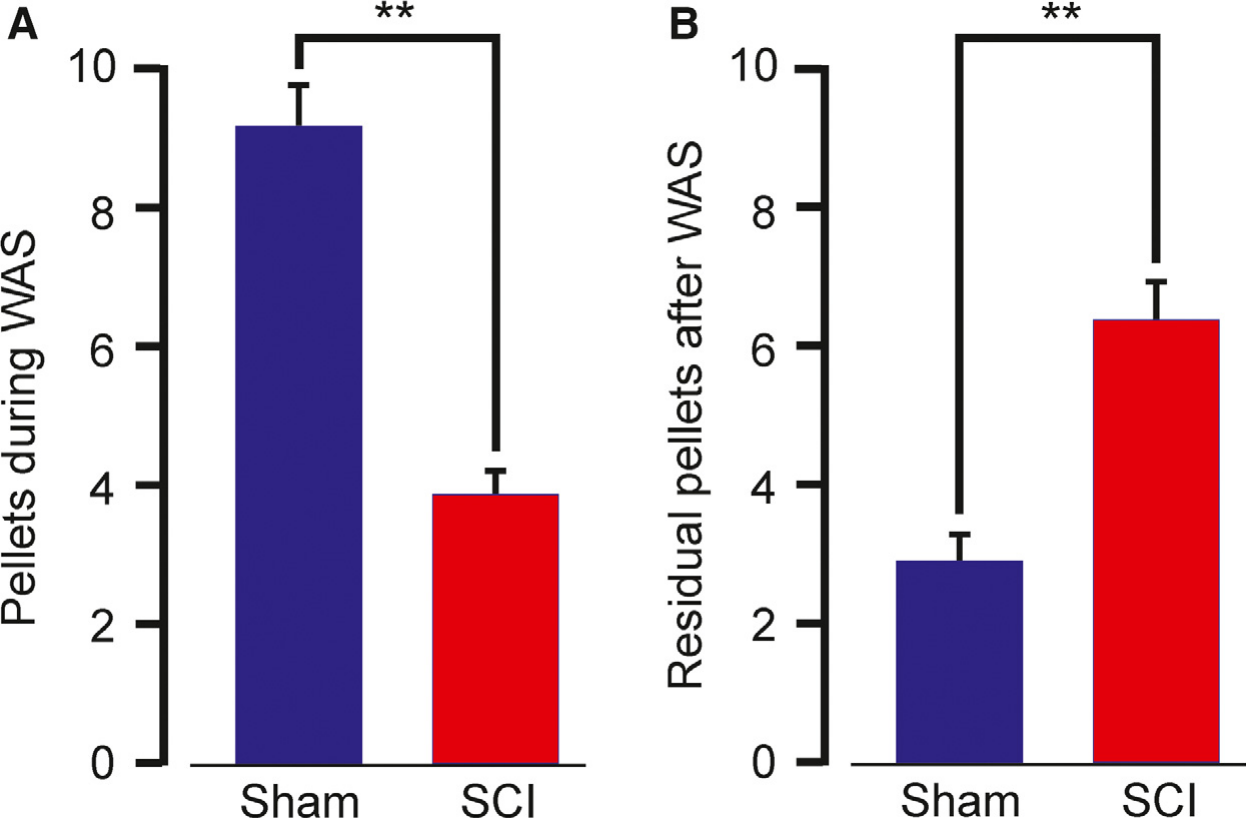
Reduced fecal pellet expulsion triggered by water avoidance and greater fecal retention in rats with spinal cords cut. (A) The numbers of fecal pellets that rats produced when placed on a platform surrounded by water for 60 min, and, (B) the residual numbers of pellets in the distal colon after 60 min on the platform are compared for rats with intact spinal cord (sham operated) and for rats with the spinal cord severed at T4 (SCI), 5 weeks before water avoidance test. Data expressed as mean ± SEM. Pellets during WAS, sham, *n* = 10, spinal lesion, *n* = 9; residual pellets, sham, *n* = 10, spinal lesion, *n* = 8. ** = significantly different *P* < 0.05.

Rats that were placed on a platform surrounded by water defecated soon after. The numbers of pellets produced in the first 15 min were fewer in rats that had their spinal cords severed at the 4th thoracic vertebrum, 5 weeks before being placed on the platform, compared with sham operated rats (Fig. 4). Moreover, the numbers of residual pellets in the colorectum 60 min after the water avoidance test were greater in spinal lesioned rats. This indicates that spinal cord transection interrupts the pathways from the cortex that are activated by water avoidance to evoke defecation.

#### Changes in colorectal sensitivity to a ghrelin receptor agonist

**Figure 5:**
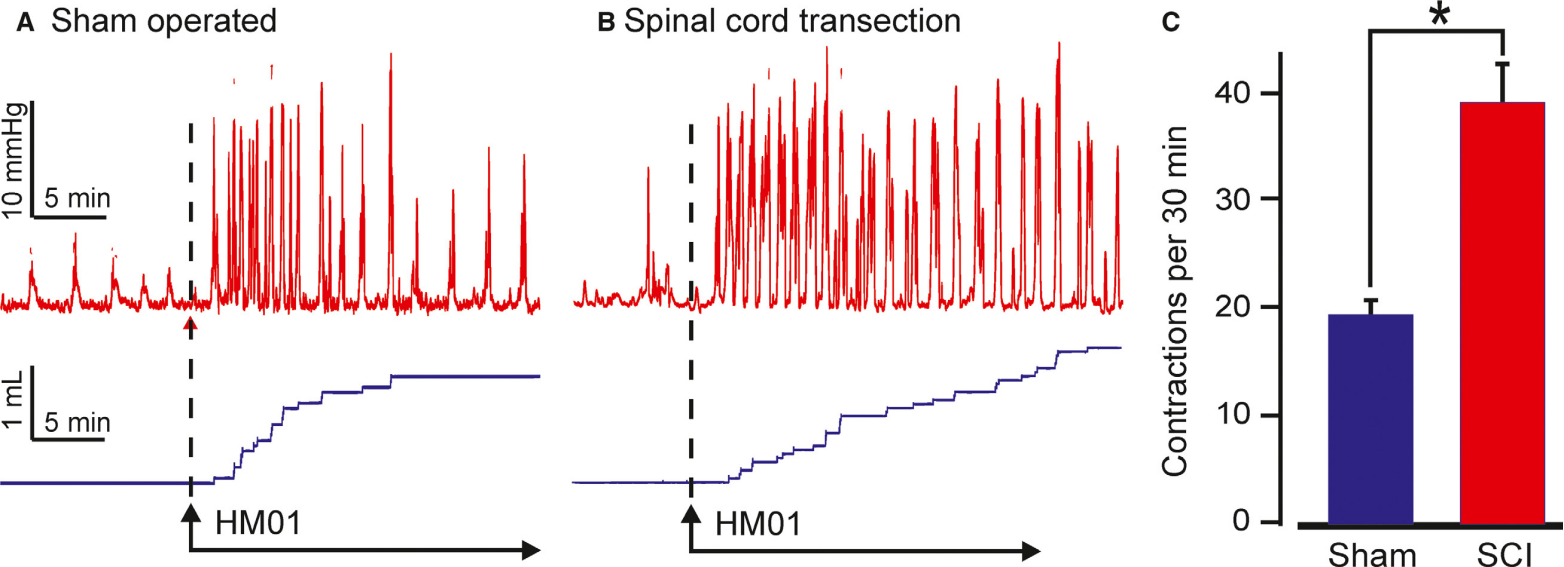
Differences in colorectal sensitivity to the GHSR1a agonist, HM01, between sham operated rats, and rats in which the spinal cord was severed at T4, 5 weeks before the experiments. The numbers of contractions with amplitudes greater than 6 mmHg are plotted. Data expressed as mean ± SEM. Sham, *n* = 9, spinal lesion, *n* = 8. * = significantly different, *P* < 0.05.

Colorectal contractile activity was measured under anesthesia in rats whose spinal cords had been severed 5 weeks previously or had sham spinal cord surgery. The rats were injected intravenously with the GHSR1a agonist, HM01 (1.0 mg·kg^−1^). This elicited propulsive contractile waves in the colorectum that expelled fluid contents (Fig. 5). The responses in rats with spinal cord transection were about twice those of rats with sham surgery (Fig. 5).

### Effect of a ghrelin receptor antagonist in ghrelin‐deficient rats

**Figure 6:**
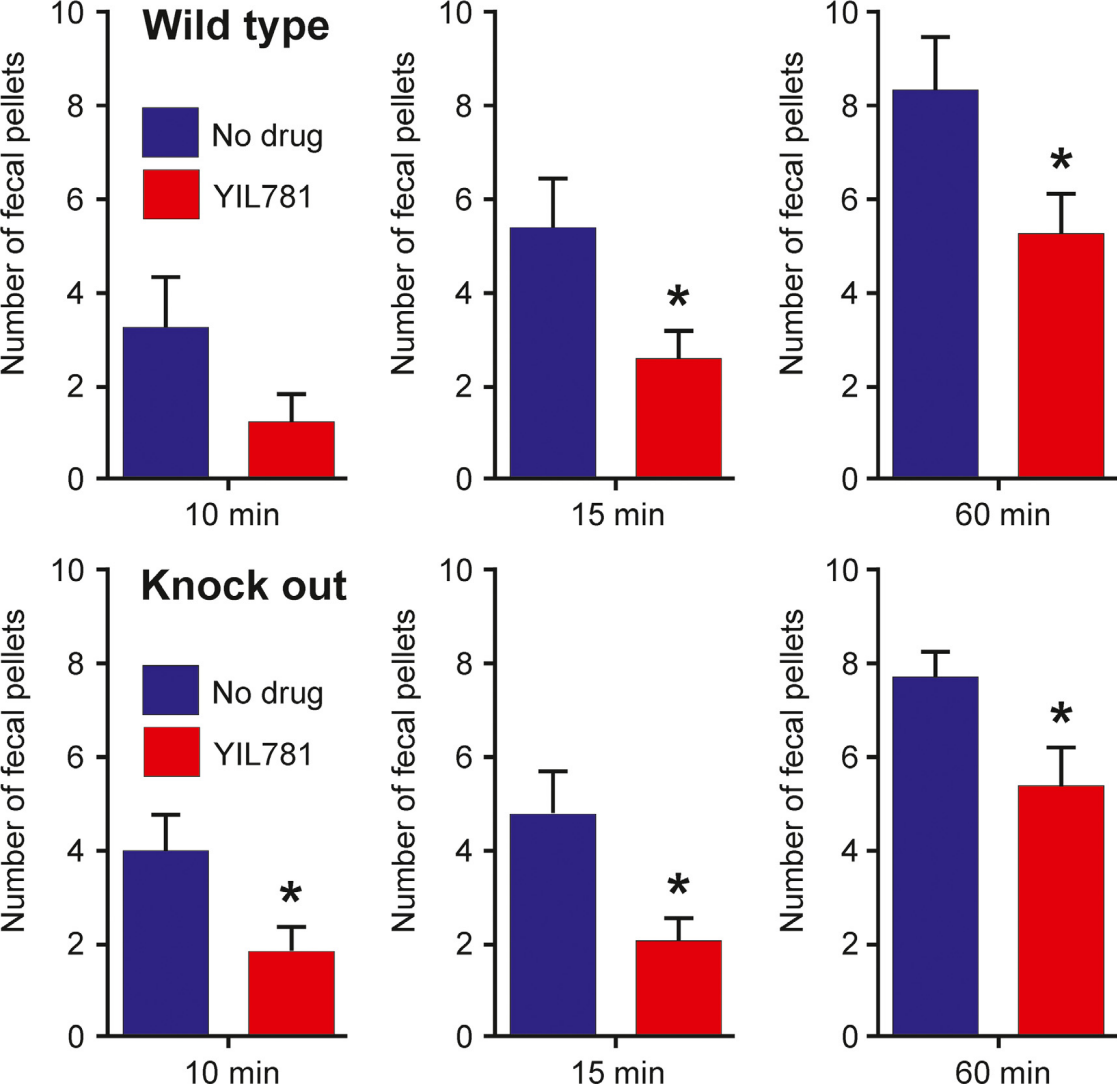
Effects of the ghrelin receptor antagonist, YIL781, on the numbers of fecal pellets produced in response to water avoidance stress. YIL781, 3 mg·kg^−1^, i.p., was injected into the rats 10 min before they were placed on a platform surrounded by water. Control rats (No drug) were injected with vehicle. YIL781 caused significant reductions in fecal output (*P* < 0.05). Data are mean ± SEM,* n* = 7 rats.

The ghrelin receptor antagonist, YIL781 (3 mg·kg^−1^, intraperitoneal) caused a similar reduction in fecal output in the wild‐type and ghrelin‐deficient rats (Fig. 6). The reduction in fecal output in the first 10 min after placing rats on the platform surrounded by water was greater than 50%, the reduction in the first 15 min, about 50% and in the hour after being placed on the platform, about 25%. We interpret this diminished effect over time to the metabolism and clearance of YIL781 after a single injection.

## Discussion

A problem that has concerned physiologists who use drugs as tools is whether the response that is observed is “pharmacological” or whether it indicates an underlying physiological relevance of the target of the drug? In the case of the effects of centrally penetrant ghrelin receptor agonists that stimulate defecation, the compounds used have been synthetic, nonnatural compounds. Thus, in the current study, we utilized a natural stimulus of defecation, water avoidance stress, and investigated whether it activated ghrelin receptors. This is a practical test in rats, but not in mice. We had previously investigated in detail whether ghrelin is present in the CNS of mice, and found that it was not present (Furness et al. 2011). However, in digestive physiology, there are considerable differences between species, including even that a peptide that has physiological importance in human, motilin, is completely absent in rat and mouse (Furness et al. 2015). Thus, we have repeated the detailed studies on the ghrelin presence using the rat, and found that it is also absent from the CNS of this species. Because it is difficult to absolutely prove a negative (the lack of ghrelin in the spinal cord of wild‐type rats) we knocked out the ghrelin gene in the rat and compared effects of a ghrelin receptor antagonist in ghrelin‐deficient and wild‐type rats. The work shows, as discussed in more detail below, that the effects of synthetic agonists of the ghrelin receptor does reflect a physiological role of the ghrelin receptor in control of colorectal function.

In this study, we found that no ghrelin could be detected by immunohistochemistry in nerve terminals of the lumbosacral spinal cord in the rat, where physiological studies have shown that ghrelin receptor agonists act to stimulate propulsive contractions of the colorectum and increase fecal output (Shimizu et al. 2006; Shafton et al. 2009; Hirayama et al. 2010; Pustovit et al. 2014; Naitou et al. 2015). We used three antibodies to the acylated form of ghrelin, the form with physiological activity at the lumbosacral spinal cord (Hirayama et al. 2010) and an antibody to the unacylated form (desacyl ghrelin). All four antibodies revealed immunoreactive enteroendocrine cells in the stomach, the body's major source of ghrelin, but none revealed immunoreactivity in the spinal cord, even when the concentrations of antibodies used for localization were increased up to 10‐fold. The immunoreactivity of gastric EEC was abolished when the ghrelin gene was disrupted, indicating that the antibodies specifically recognize ghrelin. Neuropeptides are found in high concentrations in nerve terminals and are generally very easy to locate using suitable antibodies (Ljungdahl et al. 1978; Hökfelt et al. 1984; Boyer et al. 1994; Ekblad et al. 1996). To test the preservation of neuropeptides in nerve terminals, we used antibodies to neuropeptide Y (NPY). Strong immunoreactivity for NPY was observed in the sections of spinal cord. Thus, four antibodies that are proven to recognize the acylated and unacylated forms of ghrelin in the rat did not reveal immunoreactivity in the spinal cord. We used anti‐neuronal NOS to specifically investigate the autonomic nuclei (IML) where the ghrelin receptor expressing neurons that are deduced to be the targets of ghrelin receptor agonist are located (Ferens et al. 2010a,b). No ghrelin immunoreactivity was in the IML. We used a ghrelin‐specific ELISA to seek ghrelin in extracts of the lumbosacral spinal cord. There was a low level of binding in the assay that was not different between extracts of wild‐type and ghrelin knockout spinal cord, hypothalamus or lower brain stem. We conclude that ghrelin is absent from the lumbosacral spinal cord. These data, although focused on the caudal spinal cord, are consistent with previous studies that indicate that ghrelin is absent from the CNS (Sakata et al. 2009; Furness et al. 2011; Kern et al. 2014; Cabral et al. 2017).

We next investigated whether the ghrelin receptors in the lumbosacral spinal cord might nevertheless be involved in physiological responses. We used water avoidance stress (WAS) that acts within the brain to initiate defecation (Million et al. 2000). It has been presumed that WAS communicates to the colorectum via descending spinal cord pathways. To test this, we applied WAS to rats that had their spinal cords severed 5 weeks before. WAS caused about half the number of fecal pellets to be released in 60 min from the spinal cord severed compared with sham rats. Moreover, the spinal cord transected rats had more than twice the number of pellets that remained in the colon at 60 min after WAS. This indicates that there was a deficit in the integrity of the defecation pathways that are activated by WAS when the spinal cord was severed at the lower thoracic level. However, HM01, a centrally penetrant ghrelin agonist that activates the defecation center (Naitou et al. 2015), was effective after the spine was severed. In fact, the responsiveness to HM01 was approximately doubled. One interpretation of this change is that there was a denervation sensitivity, that is, the ghrelin receptors on neurons in the lumbosacral spinal cord were lacking their normal synaptic input after the cord was severed. Denervation hypersensitivity is a well described phenomenon that occurs in both autonomic and somatic pathways when synaptic inputs are removed for days or weeks (Kuffler et al. 1971; Ekström and Wahlestedt 1982; Fujiwara et al. 1984). This implies that the ghrelin receptors are physiologically involved in the control of defecation. In a further experiment we investigated the sensitivity of the defecation response to the ghrelin receptor antagonist, YIL781. This compound, formerly named LXG934, is a centrally penetrant antagonist of the ghrelin receptor, GHSR1a, with nanomolar potency (Esler et al. 2007; Sim et al. 2014). YIL781 caused significant, and similar, reductions in the fecal output in response to WAS in both wild‐type rats and in rats lacking ghrelin. The data indicate that defecation has dependency on the ghrelin receptor, GHSR1a, but is not dependent on ghrelin.

Our observation that WAS caused expulsion of some pellets after spinal cord transection deserves comment. After the transection, the number expelled was reduced, on average, from 9 to just under 4 pellets in 60 min (Fig 4A), despite many pellets remaining in the colorectum (Fig 4B). Only about one pellet would be expected to be spontaneously released in this period. An action of stress hormones provides a possible explanation of this spinal cord independent defecation. Peripheral injection of the stress hormone, corticotrophin releasing factor (CRF), mimics stress‐induced increases in colonic transit and nonselective CRF antagonists inhibit stress‐induced stimulation of colon motility (Taché et al. 2004). An effect through long vagal pathways is also possible, as vagal nerves reach the colon in the rat (Berthoud et al. 1991). However, we are not aware of any physiological data that supports a role of vagal pathways in the colorectal response to stress.

As discussed above, the data indicate that GHSR1a is involved in the spinal pathways of defecation control. It is unknown whether GHSR1a is also involved the pathways that are activated by WAS after spinal transection. If GHSR1a is not involved in these other pathways, the results suggest that ghrelin receptor antagonism blocks most of the effect that is mediated through the spinal pathways and the defecation center.

Previous investigation has similarly identified a functional role of GHSR1a that is independent of ghrelin, in this case, the anorexic effect of dopamine was found to depend on GHSR1a (Kern et al. 2012, 2014). These authors found that GHSR1a forms a heteromer with the dopamine D2 receptor (DRD2). They identified GHSR1a:DRD2 heteromers in a subset of hypothalamic neurons. In wild‐type mice and ghrelin‐deficient mice, a DRD2 agonist elicited anorexia, but this effect was lost if GHSR1a was knocked out. Moreover, the GHSR1a antagonist blocked the anorexogenic effect of the DRD2 agonist. These observations resemble those of our investigation. It is thus pertinent that dopamine causes defecation by an action at D2 receptors in the lumbosacral defecation center of the spinal cord of the rat (Naitou et al. 2016). An alternative explanation is that there is a neurotransmitter other than ghrelin that is in descending pathways that acts on GHSR1a.

## Conflict of Interest

None declared.

## References

[phy213385-bib-0001] Acosta, A. , M. Camilleri , I. Busciglio , A. Boldingh , A. D. Nelson , and D. Burton . 2016 Short‐term effects of relamorelin on descending colon motility in chronic constipation: a randomized, controlled trial. Dig. Dis. Sci. 61:852–860. 26467700 10.1007/s10620-015-3876-5

[phy213385-bib-0002] Berthoud, H.‐R. , N. R. Carlson , and T. L. Powley . 1991 Topography of efferent vagal innervations of the rat gastrointestinal tract. Am. J. Physiol. Regul. Integr. Comp. 260:R200–R207. 10.1152/ajpregu.1991.260.1.R200 1992820

[phy213385-bib-0003] Boyer, P.‐A. , A. Trembleau , V. Leviel , and M. Arluison . 1994 Effects of intranigral injections of colchicine on the expression of some neuropeptides in the rat forebrain: an immunohistochemical and in situ hybridization study. Brain Res. Bull. 33:541–560. 7514485 10.1016/0361-9230(94)90081-7

[phy213385-bib-0004] Cabral, A. , E. J. López Soto , E. Epelbaum , and M. Perelló . 2017 Is ghrelin synthesized in the central nervous system? Int. J. Mol. Sci. 18:638–656. 10.3390/ijms18030638 PMC5372651 28294994

[phy213385-bib-0005] Charoenthongtrakul, S. , D. Giuliana , K. A. Longo , E. K. Govek , A. Nolan , S. Gagne , et al. 2009 Enhanced gastrointestinal motility with orally active ghrelin receptor agonists. J. Pharmacol. Exp. Ther. 329:1178–1186. 19252061 10.1124/jpet.108.150193

[phy213385-bib-0006] Ejskjaer, N. , G. Dimcevski , J. Wo , P. M. Hellström , L. C. Gormsen , I. Sarosiek , et al. 2010 Safety and efficacy of ghrelin agonist TZP‐101 in relieving symptoms in patients with diabetic gastroparesis: a randomized, placebo‐controlled study. Neurogastroenterol. Motil. 22:1069e281. 20524987 10.1111/j.1365-2982.2010.01519.x

[phy213385-bib-0007] Ekblad, E. , H. Mulder , and F. Sundler . 1996 Vasoactive intestinal peptide expression in enteric neurons is upregulated by both colchicine and axotomy. Regul. Pept. 63:113–121. 8837219 10.1016/0167-0115(96)00028-6

[phy213385-bib-0008] Ekström, J. , and C. Wahlestedt . 1982 Supersensitivity to substance P and physalaemin in rat salivary glands after denervation or decentralization. Acta Physiol. Scand. 115:437–446. 6184951 10.1111/j.1748-1716.1982.tb07102.x

[phy213385-bib-0009] Ellis, A. G. , P. T. Zeglinski , D. J. Brown , A. G. Frauman , M. Millard , and J. B. Furness . 2015 Pharmacokinetics of the ghrelin agonist capromorelin in a single ascending dose Phase‐I safety trial in spinal cord‐injured and able‐bodied volunteers. Spinal Cord 53:103–108. 25448190 10.1038/sc.2014.218

[phy213385-bib-0010] Esler, W. P. , J. Rudolph , T. H. Claus , W. Tang , N. Barucci , S.‐E. Brown , et al. 2007 Small‐molecule ghrelin receptor antagonists improve glucose tolerance, suppress appetite, and promote weight loss. Endocrinology 148:5175–5185. 17656463 10.1210/en.2007-0239

[phy213385-bib-0011] Ferens, D. M. , L. Yin , R. Bron , B. Hunne , K. Ohashi‐Doi , G. J. Sanger , et al. 2010a Functional and in situ hybridisation evidence that preganglionic sympathetic vasoconstrictor neurons express ghrelin receptors. Neuroscience 166:671–679. 20060438 10.1016/j.neuroscience.2010.01.001

[phy213385-bib-0012] Ferens, D. M. , L. Yin , K. Ohashi‐Doi , M. Habgood , R. Bron , J. A. Brock , et al. 2010b Evidence for functional ghrelin receptors on parasympathetic preganglionic neurons of micturition control pathways in the rat. Clin. Exp. Pharmacol. Physiol. 37:926–932. 20497419 10.1111/j.1440-1681.2010.05409.x

[phy213385-bib-0013] Fujiwara, M. , H. Hayashi , I. Muramatsu , and N. Ueda . 1984 Supersensitivity of the rabbit iris sphincter muscle induced by trigeminal denervation: the role of substance P. J. Physiol. 350:583–597. 6205140 10.1113/jphysiol.1984.sp015219 PMC1199287

[phy213385-bib-0014] Furness, J. B. , M. Costa , I. L. Gibbins , I. J. Llewellyn Smith , and J. R. Oliver . 1985 Neurochemically similar myenteric and submucous neurons directly traced to the mucosa of the small intestine. Cell Tissue Res. 241:155–163. 3839715 10.1007/BF00214637

[phy213385-bib-0015] Furness, J. B. , B. Hunne , N. Matsuda , L. Yin , D. Russo , I. Kato , et al. 2011 Investigation of the presence of ghrelin in the central nervous system of the rat and mouse. Neuroscience 193:1–9. 21835225 10.1016/j.neuroscience.2011.07.063

[phy213385-bib-0016] Furness, J. B. , J. J. Cottrell , and D. M. Bravo . 2015 Comparative physiology of digestion. J. Anim. Sci. 93:485–491. 26020739 10.2527/jas.2014-8481

[phy213385-bib-0600] Herbison, A. E. , S. X. Simonian , P. J. Norris , and P. C. Emson . 1996 Relationship of neuronal nitric oxide synthase immunoreactivity to GnRH neurons in the ovariectomized and intact female rat. J. Neuroendocrinol. 8:73–82. 8932739 10.1111/j.1365-2826.1996.tb00688.x

[phy213385-bib-0017] Hirayama, H. , T. Shiina , T. Shima , H. Kuramoto , T. Takewaki , J. B. Furness , et al. 2010 Contrasting effects of ghrelin and des‐acyl ghrelin on the lumbo‐sacral defecation center and regulation of colorectal motility in rats. Neurogastroenterol. Motil. 22:1124–1131. 20584261 10.1111/j.1365-2982.2010.01553.x

[phy213385-bib-0018] Hökfelt, T. , O. Johansson , and M. Goldstein . 1984 Chemical anatomy of the brain. Science 225:1326–1334. 6147896 10.1126/science.6147896

[phy213385-bib-0019] Kern, A. , R. Albarran‐Zeckler , H. E. Walsh , and R. G. Smith . 2012 Apo‐ghrelin receptor forms heteromers with DRD2 in hypothalamic neurons and is essential for anorexigenic effects of DRD2 agonism. Neuron 73:317–332. 22284186 10.1016/j.neuron.2011.10.038 PMC3269786

[phy213385-bib-0020] Kern, A. , C. Grande , and R. G. Smith . 2014 Apo‐ghrelin receptor (apo‐GHSR1a) regulates dopamine signaling in the brain. Front. Endocrinol. 5:129–136. 10.3389/fendo.2014.00129 PMC4135303 25183960

[phy213385-bib-0021] Kuffler, S. W. , M. J. Dennis , and A. J. Harris . 1971 The development of chemosensitivity in extrasynaptic areas of the neuronal surface after denervation of parasympathetic ganglion cells in the heart of the frog. Proc. R. Soc. Lond. B Biol. Sci. 177:555–563. 4396520 10.1098/rspb.1971.0047

[phy213385-bib-0022] Ljungdahl, A. , T. Hökfelt , and G. Nilsson . 1978 Distribution of substance P‐like immunoreactivity in the central nervous system of the rat‐I. Cell bodies and nerve terminals. Neuroscience, 1:861–943. 10.1016/0306-4522(78)90116-1 366451

[phy213385-bib-0023] Million, M. , L. Wang , V. Martinez , and Y. Taché . 2000 Differential Fos expression in the paraventricular nucleus of the hypothalamus, sacral parasympathetic nucleus and colonic motor response to water avoidance stress in Fischer and Lewis rats. Brain Res. 877:345–353. 10986349 10.1016/s0006-8993(00)02719-0

[phy213385-bib-0024] Mizutani, M. , K. Atsuchi , A. Asakawa , N. Matsuda , M. Fujimura , A. Inui , et al. 2009 Localization of acyl ghrelin‐ and des‐acyl ghrelin‐immunoreactive cells in the rat stomach and their responses to intragastric pH. Am. J. Physiol. Gastrointest. Liver Physiol. 297:G974–G980. 20501445 10.1152/ajpgi.00147.2009

[phy213385-bib-0025] Naitou, K. , T. P. Mamerto , R. V. Pustovit , B. Callaghan , L. R. Rivera , A. J. Chan , et al. 2015 Site and mechanism of the colokinetic action of the ghrelin receptor agonist, HM01. Neurogastroenterol. Motil. 27:1596–1603. 10.1111/nmo.12688 26416336

[phy213385-bib-0026] Naitou, K. , H. Nakamori , T. Shiina , A. Ikeda , Y. Nozue , Y. Sano , et al. 2016 Stimulation of dopamine D2‐like receptors in the lumbosacral defaecation centre causes propulsive colorectal contractions in rats. J. Physiol. 594:4339–4350. 26999074 10.1113/JP272073 PMC4967751

[phy213385-bib-0027] Patterson, M. , K. G. Murphy , C. W. le Roux , M. A. Ghatei , and S. R. Bloom . 2005 Characterization of ghrelin‐like immunoreactivity in human plasma. J. Clin. Endocrinol. Metab. 90:2205–2211. 15657369 10.1210/jc.2004-1641

[phy213385-bib-0028] Pustovit, R. V. , B. Callaghan , S. Kosari , L. R. Rivera , H. Thomas , J. A. Brock , et al. 2014 The mechanism of enhanced defecation caused by the ghrelin receptor agonist, ulimorelin. Neurogastroenterol. Motil. 26:264–271. 24304447 10.1111/nmo.12259

[phy213385-bib-0029] Pustovit, R. V. , J. B. Furness , and L. R. Rivera . 2015 A ghrelin receptor agonist is an effective colokinetic in rats with diet‐induced constipation. Neurogastroenterol. Motil. 27:610–617. 25616061 10.1111/nmo.12517

[phy213385-bib-0030] Sakata, I. , Y. Nakano , S. Osborne‐Lawrence , S. A. Rovinsky , C. E. Lee , M. Perello , et al. 2009 Characterization of a novel ghrelin cell reporter mouse. Regul. Pept. 155:91–98. 19361544 10.1016/j.regpep.2009.04.001 PMC2697121

[phy213385-bib-0031] Shafton, A. D. , G. J. Sanger , J. Witherington , J. D. Brown , A. Muir , S. Butler , et al. 2009 Oral administration of a centrally acting ghrelin receptor agonist to conscious rats triggers defecation. Neurogastroenterol. Motil. 21:71–77. 18694442 10.1111/j.1365-2982.2008.01176.x

[phy213385-bib-0032] Shimizu, Y. , E. C. Chang , A. D. Shafton , D. M. Ferens , G. J. Sanger , J. Witherington , et al. 2006 Evidence that stimulation of ghrelin receptors in the spinal cord initiates propulsive activity in the colon of the rat. J. Physiol. 576:329–338. 16873401 10.1113/jphysiol.2006.116160 PMC1995628

[phy213385-bib-0033] Sim, Y.‐B. , S.‐H. Park , S.‐S. Kim , C.‐H. Kim , S.‐J. Kim , S.‐M. Lim , et al. 2014 Ghrelin administered spinally increases the blood glucose level in mice. Peptides 54:162–165. 24472858 10.1016/j.peptides.2014.01.015

[phy213385-bib-0034] Taché, Y. , V. Martinez , L. Wang , and M. Million . 2004 CRF_1_ receptor signaling pathways are involved in stress‐related alterations of colonic function and viscerosensitivity: implications for irritable bowel syndrome. Br. J. Pharmacol. 141:1321–1330. 15100165 10.1038/sj.bjp.0705760 PMC1574904

[phy213385-bib-0035] Williamson, S. , S. Pompolo , and J. B. Furness . 1996 GABA and nitric oxide synthase immunoreactivities are colocalized in a subset of inhibitory motor neurons of the guinea‐pig small intestine. Cell Tissue Res. 284:29–37. 8601294 10.1007/s004410050564

